# Reconstruction of Autobiographical Memories of Violent Sexual-Affective Relationships Through Scientific Reading on Love: A Psycho-Educational Intervention to Prevent Gender Violence

**DOI:** 10.3389/fpsyg.2018.01996

**Published:** 2018-10-24

**Authors:** Sandra Racionero-Plaza, Leire Ugalde-Lujambio, Lídia Puigvert, Emilia Aiello

**Affiliations:** ^1^Department of Psychology, Universidad Loyola Andalucía, Seville, Spain; ^2^Department of Educational Organization and Didactics, University of the Basque Country, Bilbao, Spain; ^3^Department of Sociological Theory, University of Barcelona, Barcelona, Spain; ^4^Affiliated Member of the Centre for Community, Gender and Social Justice, Institute of Criminology, University of Cambridge, Cambridge, United Kingdom; ^5^Community of Researchers on Excellence for All (CREA), University of Barcelona, Barcelona, Spain

**Keywords:** gender violence, prevention, autobiographical memory, reading, youth, successful actions, effective interventions

## Abstract

Violence in sexual-affective relationships among teens and young people is recognized as a social, educational, and health problem that has increased worldwide in recent years. Educational institutions, as central developmental contexts in adolescence, are key in preventing and responding to gender violence through implementing successful actions. In order to scientifically support that task, the research reported in this article presents and discusses a psycho-educational intervention focused on autobiographical memory reconstruction that proved to be successful in raising young women’s critical consciousness about the force of the coercive discourse upon sexual-affective experiences and memories. We examined among a sample of young women (*n* = 32, age range 17–30) whether reading a scholarly text about love, the *Radical Love* book, modified autobiographical memories of violent sexual-affective relationships in line with preventing future victimization. This group was compared with a control group (*n* = 31, age range 17–30). Memory reports were collected before and after the reading and coded to analyze their content, both quantitatively and qualitatively. Memory quality features were assessed with the Memory Quality Questionnaire (MMQ). A focus group was also conducted to examine the personal impact of the intervention on participants. Compared with controls, the experimental group had stronger critical memories (of episodes involving violence), an average decrease in positive emotions induced by recall, and an average increase in negative emotions. The results show the effectiveness of the reading intervention designed in relation to gender violence prevention, as they indicate the ability of the psycho-educational action to debilitate the force of the coercive discourse in young women’s memories. The findings both advance knowledge on the reconstructive nature of autobiographical memories of violent sexual-affective relationships in female youth and indicate the potential of memory-based interventions as an instrument to prevent and reduce gender violence in school contexts. Teachers and teaching staff, and educational psychologists, among others, can benefit from these results by expanding the tools they have to address gender violence among female adolescents and youth.

## Introduction

Violence in sexual-affective relationships among teens and young people is recognized as a social and health problem that has increased worldwide in recent years. Data indicate that 32% of women in North America, 38% in Latin America and the Caribbean, 46% in Europe, 64% in Africa, 67% in Asia, and 68% in Oceania have experienced intimate partner violence (IPV) at least once in their lifetime (United Nations Statistics Division, 2015). Thirty percentage of young females aged 15–19 are victims of violence in their sexual-affective relationships (World Health Organization, 2015), and this abuse often begins in preadolescence (Banyard and Cross, 2008; Leen et al., 2013).

A study on the prevalence of sexual aggression among young people in 10 European countries (Austria, Belgium, Cyprus, Greece, Lithuania, the Netherlands, Poland, Portugal, Slovakia, and Spain) found that between 19.7 and 52.2% of females aged 18–27 reported having experienced at least one incident of sexual victimization (Krahé et al., 2015). Along this line, the European Union Agency for Fundamental Rights (2014) estimated that 35% of European women age 15 or older are victims of physical and sexual violence. In a sample of women aged 15–22 from Sweden, 21% reported having been psychologically, sexually, and physically abused. This study also shows that this victimization is related to suffering sexually transmitted diseases (Blom et al., 2016). In Germany, in a sample of teens aged 14–18, 65.7% of females declared that they had suffered at least one type of disrespectful behavior or violence, 61% emotionally difficult situations, 26% unwanted sexual behavior, and 11% physical violence. In this same study, adolescents who were victims of partner abuse had a lower quality of life compared with female teenagers who were not victimized (Blattner et al., 2015). In the United States, in a sample of adolescents aged 14–21, 51% of females reported being victims of at least one type of abuse: psychological, physical or sexual (Ybarra et al., 2016). In another study conducted in the United States, 22% of adult female victims of rape reported that their first experience of IPV occurred when they were between 11 and 17 years old (Black et al., 2011). In Canada, the Enquête Sociale Générale found that 37% of women had been sexually assaulted, 71% of whom were between 15 and 24 years old (Statistique Canada, 2015). Importantly, several studies have shown that IPV at a young age occurs in both stable and sporadic sexual-affective relationships and among women with different socioeconomic statuses (Trygged et al., 2014; Bay-Cheng and Bruns, 2016). The consequences of this violence in adolescence and youth are numerous and introduce major obstacles for positive development.

At the psychological level, young women may experience symptoms of depression and anxiety (Ackard et al., 2007; Exner-Cortens et al., 2013), post-traumatic stress disorders (Wolitzky-Taylor et al., 2008), suboptimal psychosocial functioning, impaired self-esteem (Chiodo et al., 2012), and suicidal ideation (Ely et al., 2011; Exner-Cortens et al., 2013), as well as cognitive and emotional damage, which influences their long-term development (Ackard and Neumark-Sztainer, 2002).

Research has also shown that victims of IPV may engage in unhealthy behaviors, such as using tobacco, drugs and alcohol, and may exhibit antisocial behaviors (Roberts et al., 2003; Exner-Cortens et al., 2013; Foshee et al., 2013; Gilmore et al., 2016) and eating disorders (Ackard and Neumark-Sztainer, 2002; Sharp and Keyton, 2016). Additionally, many of those adverse consequences may extend into adulthood and increase the likelihood of establishing violent relationships later in life (Sunday et al., 2011; Turanovic and Pratt, 2015). At the educational level, evidence demonstrates an increase in the dropout rate and a decline in academic performance among victims of gender violence (Banyard and Cross, 2008; Holmes and Sher, 2013). In addition, these women are at higher risk for victimization during college (Smith et al., 2003).

A large number of studies have focused on investigating the causes, risk factors, and predictors of gender violence (Sheridan and Lyndon, 2012; Karlsson et al., 2016; Kast et al., 2016). Among those many series of factors, evidence supports the existence of a *coercive discourse* (Puigvert and Flecha, 2018), a discourse which, shaped by an imbalance in power within relationships, influences socialization into linking attractiveness to people with violent attitudes and behaviors, while non-violent people and relationships are – because of this coercive discourse – mostly perceived as convenient but not exciting. Importantly, the scientific examination of this coercive discourse indicates that its implicit pattern of attraction may be learned through direct and/or indirect experience throughout the lifespan, adolescence being a key period in which such learning can occur (Gómez, 2015). In the vast majority of media, movies, songs, video clips, TV shows, youth literature, and Internet forums, the male characters presented as most attractive and successful have dominant, aggressive, and sexist behaviors and attitudes toward women (Gómez, 2015). This can later affect some young women’s sexual-affective preferences and choices (Rebellon and Manasse, 2004; McDaniel, 2005; Montañés et al., 2013), talking then about *coerced preferences* (Puigvert and Flecha, 2018), i.e., preferences which are coercively shaped and driven by the existing dominant discourse. Likewise, research has shown that dialogs among friends can create expectations about behavior and gender in relation to the aggressive behavior of men (Kimmel, 1996; Giordano, 2003), so conversations within peer groups are likely to be shaped by the coercive discourse and might reproduce it. Yet the fact that the coercive discourse, and different kinds of masculinities and emotions (Ramírez Rodriguez et al., 2017) have been socially constructed opens up the possibility for the subjects to enact their own agency and free choice, and modify the influence of such discourse (Gómez, 2015; Puigvert, 2016).

The gender studies literature has indicated the importance of examining the self-experience of violent intimate relationships to better understand the socialization process in this area for every individual (Oliver and Valls, 2004). In this exploration, the first sexual-affective experiences – in both stable and sporadic relationships – are critical because they become an important basis of interpretation and behavior in subsequent relationships (Gómez, 2015). Life narratives are central in this sense (McAdams, 1998; Bruner, 2004; Akanle, 2018); *what* we remember about our personal past – that is, our autobiographical memories (Conway and Pleydell-Pearce, 2000) - and *how* we remember it influence our sense of self, current emotions, and prospective thinking. This is due to the key psychological functions that autobiographical memories accomplish. When we share autobiographical memories with others, we create and strengthen social bonds (Conway, 2003), and based on our personal memories, we make sense of our life, make decisions, plan our future (Klein et al., 2010; Vranic et al., 2018), and, overall, develop an identity (Baddeley, 2014). In the words of Kandel (2007, p. 10): “we are who we are because of what we learn and what we remember.”

Yet not all memories are equally relevant at all stages of life. Studies in the area of autobiographical memory have shown that most accessible memories are subject to the objectives of the “self” in every developmental stage (Conway and Holmes, 2004). With regard to our study, research has indicated that among adolescents and young people, the most salient memories are linked with love and the establishment of sexual-affective relationships (Conway and Holmes, 2004), that is, relationships which involve affective and sexual experiences in the form of dating, stable relationships, “hooking up,” etc. Recalling those memories not only maintains but also fosters feelings of intimacy and connection with the person who is remembered (Alea and Bluck, 2007). When speaking about memories of violent sexual-affective relationships, the transformation of such feelings of intimacy and connection – if any – with the perpetrator of violence becomes essential in order to prevent future victimization. In spite of the ability of autobiographical memories to influence prospective thinking (Williams et al., 2008; Klein et al., 2010), emotional wellbeing, and overall health (Rubin, 2010) the study of autobiographical memories of violent sexual-affective relationships as a way to tackle violence against women from a preventive perspective has been very scarce, even more from the point of view of interventions in school settings. In regard with this last aspect, literature in educational sciences shows that much needs to and can be done.

Research in education has well established the important role of schools, as central developmental contexts, in tackling all kinds of violence, including violence against girls and young women (Fineran and Bennett, 1999; Lavoie et al., 2000; Silverman et al., 2001). The benefits of addressing violent relationships in schools go beyond solving problems in social relations, but they reach academic achievement and learning processes, as they are inseparable from students’ emotional wellbeing (Eisenbraun, 2007). Once violence decreases, learning and achievement improve. For the specific case of gender violence, school-based research has shown that it is a type of violence that is too often involved in bullying in schools (Díaz-Aguado, 2006). Nonetheless, few studies about school violence address the gender violence dimension in specific ways (Farrington, 1993; O’Moore and Minton, 2005; Banks, 2010; Yoneyama and Naito, 2010). In terms of intervention, schools are ideal settings for the implementation of preventive strategies and programs (Ozer, 2006; Lundgren and Amin, 2015), and for the case of gender violence prevention even more, as schools, high schools and colleges are among the most essential settings for socialization in youth (Theimann, 2016).

Some prevention strategies in secondary schools have proven their efficacy at an international level. One example is *Safe Dates*, an evidence-based program focused on prevention of dating violence among teens (Foshee et al., 2014). Another example is *The Fourth R*, which has shown to be able to reduce violence in teen couples, and gives the possibility to other socialization agents, such as family members and teachers, to access the intervention strategies (Wolfe et al., 2009). Nevertheless, a meta-analysis of school-based interventions addressed to prevent and reduce violence in teen dating relationships concludes that in spite of some promising results, many initiatives implemented in educational settings are not decreasing violent relationships significantly (De La Rue et al., 2017). Therefore, it is central to advance toward the identification of educational actions that evidence their impact in preventing and responding to gender violence. Out study is a contribution in this regard.

Also, despite the relevance of autobiographical memories of violent sexual-affective relationships in relation to future victimization, existing prevention programs in schools rarely deal with personal memories. In addition, interventions in high schools are mostly designed without differentiating between young women who had any experience of violent sexual-affective relationships, stable or sporadic, and young women who had not. Nonetheless, the existing data already shared in the beginning of this introduction makes clear that victims of gender violence are found among very young females, so specific intervention strategies addressing those females’ needs appear necessary.

### Current Study

Memories of violent sexual-affective relationships which, due to the coercive discourse, might include feelings of attraction and desire, place some young females at risk of victimization, given the prospective functions of such memories. Along the line of social impact of research (Reale et al., 2017; Soler-Gallart, 2017; Pulido et al., 2018), and in relation to key related findings from the Free Teen Desire project (Puigvert, 2015/2016), our study sought contributing to prevent gender violence revictimization. This task appeared promising in the light of scientific evidence from the field of memory studies.

Different from other types of human memories, autobiographical memories are malleable in nature (Cohen and Conway, 2008) - that is, they can be reconstructed through specific learning experiences and social interactions (Stone et al., 2010; Hirst and Rajaram, 2014). With regard to the topic under study here, this opens a window for memory-based interventions in schools that support the agency of young women to reconstruct memories of violent sexual-affective relationships, raising awareness on these personal experiences and changing the valence and arousal of the emotional memories toward increasing rejection of violent relationships experienced. That is the kind of intervention that we designed for the study reported in this article, an intervention which consisted of reading on love and attraction from a scientific perspective (Gómez, 2015) to support the possibility of memory recall and memory reconstruction if freely chosen by the participants in the enactment of their agency. The design of this intervention was framed by the current “social turn” in memory research (Hirst and Rajaram, 2014), which states that social interactions, social experiences, certain artefacts, and other sociocultural mediators scaffold individuals’ memory construction and reconstruction (Nelson and Fivush, 2004; Wagoner and Gillespie, 2014).

## Materials and Methods

### Participants

The original sample comprised 75 college females attending a public university in a city in northern Spain. 10 participants were removed from analysis for failing to complete the task of reading the book chapters. Among the 65 young females included in the analyses, the ages ranged from 17 to 27 years (*M* = 20.16 years, *SD* = 2.77). Participants were recruited from in-person advertisements in some of their undergraduate classes and were not students of the researchers at that time. They were informed that participation was sought “for research on autobiographical memories of sexual-affective relationships from a gender perspective.” Inclusion criteria involved self-identification as having experienced IPV - as defined by the international scientific community (Breiding et al., 2015) - to some degree in a sporadic or stable intimate relationship and not having any diagnosed memory deficit. The researcher gave examples of concrete behaviors involving physical, psychological, and sexual violence according to the international definition of IPV (Breiding et al., 2015) that could be familiar for the participants. Ethically, this supported greater reliability in the self-identification, as well as prevented from being the writing of the memory reports triggers in the self-identification.

### Material and Procedure

This research study followed all ethical standards for research involving human participants from Horizon 2020 (European Commission) as well as from the Declaration of Helsinki (World Medical Association [WMA], 2013). Before participants being involved in the study, the researchers fully informed them about the research and they completed written Informed Consent. Research participants had time to read the consent form and to ask questions to the researchers. Explanations were given by the researchers when necessary. The information provided in the consent form explained the objective of the study, the voluntary nature of participation, the possibility to withdraw from the study at any time, the procedure to collect the data, the materials and measures to be used, and the anonymity and privacy statement.

An *ad hoc* Ethics Committee was established for this specific study. The president of this Committee was Dr. Marta Soler, member of Ethical Review Panels of research projects of the European Framework Program. The other two members of the Committee were Dr. Teresa Sordé and Dr. Patricia Melgar. Teresa Sordé is evaluator of projects presented to calls of the European Research Framework Program. Patricia Melgar is founder member of the Plataforma Unitària contra les Violències de Gènere (Unitary platform against gender violence) in Catalonia (Spain). This Ethics Committee revised and approved the study.

#### Recall 1

After the study was presented and the participants responded to initial background questions, they proceeded with the memory-sharing portion of the research. This occurred in a typical classroom at the university. Female participants were asked to recall sexual-affective relationship memories with a male who had any violent attitude or behavior with them included in the international definition of IPV (Breiding et al., 2015). Specifically, participants were told that the memory could be about a sexual-affective relationship (sporadic or stable) that happened long ago or more recently, as long as the memory was accessible and related to a relationship with a man with violent behavior. The researcher also explained that it was possible that they had mixed feelings (positive and negative) or even only positive feelings toward the man and the relationship and that this was fine to report. Participants were given a few minutes to think about the relationship and events related to it, and then, they were asked to “write about where you were, what you did, and what you were thinking and feeling.”

The specific instructions were: “Now I want you to think about one or more episodes that were particularly meaningful to you in that sexual-affective relationship and your reactions to it. Try to just think freely about the event/s and the relationship, your experience of it, and what has happened since. Write down whatever comes to mind. I’m going to give you about 10 min to think about the event, your memory of it, and your reaction to it.” The experimenter allowed participants approximately 20 min to write about their memories. Their memories were probed to exhaustion. Participants wrote their memories in a computer at the university and left the document there. Later, the document was saved by the researchers in a USB and deleted from the computers, which belonged to the university. This ensured no possibility for the participants to go back to their writings.

After the reports were collected, participants completed the MMQ (Alea and Bluck, 2007).

Writing the memories in private – instead of sharing them orally in an interview – had the benefit of giving more intimacy to the female participant, as memories of violent sexual-affective experiences are personal, intimate, and highly emotional, and thus can be difficult to disclose to a researcher, someone who the participant does not know, and in a face-to-face situation. Likewise, the writing contributes to mitigate the effects of social desirability, which could manifest in loss of sincerity or less sharing of recalled memories. The writing of memory reports of traumatic and highly emotional autobiographical memories, as well as 20-min timing for the writing, is common in research designs of studies focused on this and similar topics (Alea, 2010; Harris et al., 2010).

#### Intervention

Participants were randomly assigned to an intervention group and to a control group, in which no intervention was applied. Before they left the room, participants were asked for their email addresses, and an electronic copy of selected pages (chapters 1, 3, 4, and 5) of the Spanish version (Gómez, 2004) of the book *Radical Love*. *A Revolution for the 21st Century* was sent immediately to participants in the intervention condition. They were given 10 days to read this material. No instructions were given regarding how and where to do the reading except that it must be performed individually. The timing of 10 days responds to ensuring enough time to make a comprehensive individual reading of the four chapters of the *Radical Love* book.

#### Recall 2

Exactly 10 days after the reading, the researchers emailed participants and asked them to recall the same event in their autobiographical memory that they had written about in the initial session. The instructions were, “First, please just think freely about the event/s and the relationship, your experience, and your feelings about it. Then, write your memories of the episodes (including thoughts, feelings, and experience) in a blank electronic document for 20 min (or less, if that is enough for you).” Participants were also asked to fill in a blank copy of the MMQ after they wrote down the memory report. Researchers asked participants to complete and return the memory report and the questionnaire by email as soon as possible. If participants did not respond within 2 days, the experimenters sent them a reminder email.

#### The Book *Radical Love*

*A revolution for the 21st century* (Gómez, 2015) was selected as a central stimulus in the intervention for the following reasons. First, it is a scholarly book that presents a social theory on love that states that love and attraction among humans have social roots. In this sense, the text reviews, discusses, and builds on theories on love and intimacy and elaborates the argument that patterns of attractiveness (what is considered attractive) have been constructed throughout history through social interactions and agents of socialization. In pointing to the social nature of love and attraction, the text makes clear the centrality of all subjects, of their human agency, to freely choose in the sexual and affective life, knowing the existence of coercive discourses. Second, the text includes the points of view of female and male adolescents collected from magazines, communicative life stories, and focus groups. In these texts, young participants share their thoughts about different types of male behavior and attitudes toward women and what many of these men think, demystifying any positive image of those masculinities. Third, the book employs a “language of desire” (Flecha and Puigvert, 2010), which describes reality in terms of like, desire, emotions, and feelings, in contrast to a “language of ethics,” which analyzes reality in terms of what is good and what is bad. Other research has shown that using a “language of desire” - instead of a “language of ethics” alone - is much more effective for deeply understanding and discussing the coercive discourse and, particularly, for individuals having real freedom to decide, dismantling attraction to violent masculinities (Puigvert, 2016). For the aforementioned reasons, *Radical Love* (Gómez, 2015) aids in separating attractiveness and violence.

Communicative discussion group after post-test with seven participants. Some of the participants in the intervention group asked the experimenters for a space to share their experiences of reading the book and what it had meant to them. Following the ethical conduct which is expected from researchers using the communicative methodology employed in the study (Gómez et al., 2011), the investigators responded to the participants’ demand organizing a communicative discussion group with the participants who asked for group sharing of their experience in the study. Indeed, the researchers had foreseen that this participants’ petition could arise given the emotional implications of the intervention. The final group comprised seven participants, who voluntarily nominated themselves to participate. The discussion in the focus group was centered on a broad theme: what the intervention meant to the participants and did for them. The researchers also participated in the dialog from their expert knowledge, providing support to participants when necessary.

### Measures and Instruments

#### Memory Quality

Memory quality was assessed with items from the MQQ (Alea and Bluck, 2007). Specifically, questions were employed that focused on (a) feelings and emotions induced by the recall of the memory and (b) the last time participants had shared these memories. Regarding the first set of questions, all followed the simple format “Did this memory make you feel… (circle one number for each emotion),” and participants chose among happy, sad, afraid, surprised, and angry. Responses to all questions were given on a 5-point Likert scale ranging from not at all (1) to extremely (5). Regarding the last time they had shared these memories, the question posed to participants was “When was the last time you talked about or shared this memory with others? (check all that apply),” with possible responses being “yesterday,” “a week ago,” “a month ago,” “a year ago,” “more than a year ago,” “never,” and “others, please describe.”

### Data Analysis

#### Coding of Memory Reports

The themes present in participants’ memory reports in both the intervention and control conditions were coded by the researchers in a line-by-line fashion. The early coding framework was built following a deductive flexible approach. We employed a main general category called “critical memory,” defined by Flecha et al. (2017). This theme was refined throughout the data analysis to respond to features related to that theme which emerged more strongly when the written memories were examined in detail. In particular, we used a subset (20%) of the transcripts to test the significance of the “critical memory” theme and see the potential emergence of other features relevant to this theme. The category “critical memory” included two codes and accounted for a significant number of statements given by our participants. Consensus for the coding was achieved through discussion. Discrepancies were resolved primarily by clarifying the interpretation of fragments of the written memories in relation to the definition of the main theme analyzed, along the lines that follow. According to the definition of “critical memory” (Flecha et al., 2017), text from the memory reports was coded as such when that piece of a participant’s narrative shared two subthemes at the same time: (a) Intimate Partner Violence (Code: IPV) and (b) Critical Consciousness (Code: CC). Code IPV implies explanation of episodes of IPV – as defined by the international scientific community (Breiding et al., 2015) – that described *violent episodes* and *violent details* in the stable or sporadic intimate relationship, including unwanted sexual contact, stalking acts, coercive control, defamation on social media, public humiliation, etc. Code CC indicates critical consciousness about the episode of IPV explained in the memory report (coded as IPV) as expressed through details about one’s behavior and/or feelings, and/or those of other close ones in relation to the violent episode and/or behavior and/or attitudes of the perpetrator. Sometimes, one’s feelings and behaviors are presented as analyzed through the lenses of the coercive discourse. Also, *critical consciousness* often results in making negative judgments about the violent episode and/or behavior and/or attitudes of the man. Those details work to explore and unpack the violent episode and the violent relationship at a deeper level. These memories are *critical* because they provide new insight into the memory, give new meaning to the experience and, of interest to this study, help encourage rejection of the experience and the perpetrator’s behavior and can empower the woman in relation to what occurred and to her sexual-affective future (Flecha et al., 2017).

After the category “critical memory” (Flecha et al., 2017), particularly its associated codes were refined, we individually coded all memory reports and the transcript of the focus group using NVivo software. We resolved disagreements by discussion. We identified theme frequencies and theme coverage percentage in the memory reports of participants in every condition. This served us to summarize some of the major qualitative themes generated from participants’ memory reports (Hesse-Biber, 2016). Qualitative analyses of the memory reports were also performed using the same codes – so that qualitative analyses were conducted of the same text that had been quantified – in order to understand the meanings constructed by participants and which were ascribed to critical memories. For the quantitative data, we conducted *t*-test analyses to examine whether there were differences in theme frequencies and theme coverage percentages between the pre-test and the post-test in the intervention condition (writing of memories and reading) and in the control condition (writing of memories with no reading). The same analyses were performed to explore whether there were differences between the pre-test and the post-test in every condition for results from the two selected questions of the MMQ. One of the questions examined the emotions induced by the recall of the autobiographical memories, and the other one examined the sharing of the memory with others. Data from the focus group was analyzed qualitatively searching for what the experience meant for the participants in the intervention group, focusing on personal impact. Additionally, a deductive analysis of the transcribed verbal data from the focus group was performed examining presence of “critical memories” in the group discussion. Contrasting this analysis of data from the focus group with data from the memory reports, which had been analyzed quantitatively and qualitatively, allowed raising trustworthiness (Lincoln and Guba, 1985), as well as it informed about consistency of the codes employed (Hesse-Biber, 2016).

## Results

### Critical Memories

The participants who read *Radical Love* experienced a significant increase in critical memories related to the sexual-affective relationship shared in the memory report (see Table 1). This implied recalling and disclosing more episodic details of the relationship related to disdain, humiliation, and other IPV characteristics, accompanied by self-consciousness.

**Table 1:** Pre- and post-test critical memories.

	Experimental group (*n* = 32)				Control group (*n* = 31)			
		
Theme	Pre-mean	SD	Post-mean	SD	Pre-mean	SD	Post-mean	SD
Coverage^a^	15.75	14.86	30.72	18.4	9.97	3.67	11.00	7.2
Reference^a^	2.63	1.69	4.04	2.49	2.11	2.26	1.82	1.33


*^*a*^Experimental group increased significantly after the intervention.*

**FIGURE 1:**
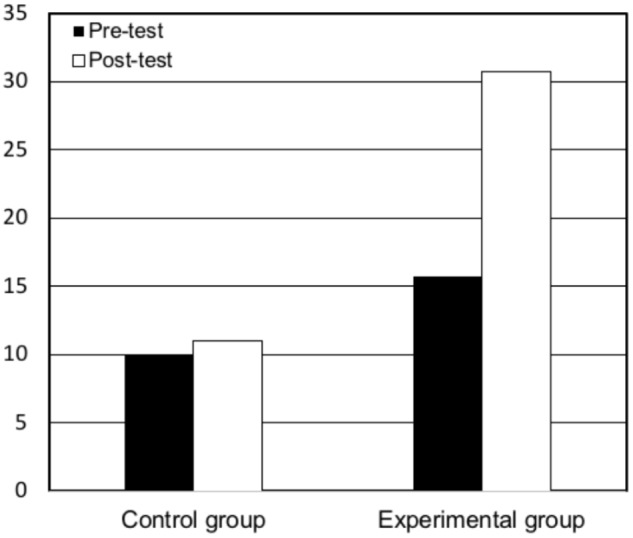
Pre- and post-test mean of coverage of critical memories by condition.

**FIGURE 2:**
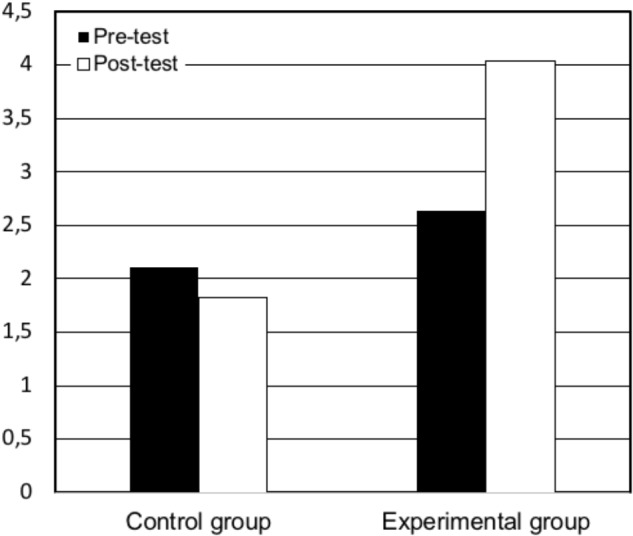
Pre- and post-test mean of reference to critical memories by condition.

The increase in critical memories occurred for both theme coverage *t*(25) = 3.65, *p* < 0.05, Cohen’s *d* = -0.91 (see Figure 1), and theme reference *t*(25) = 2.19 *p* < 0.05, Cohen’s *d* = -0.66 (see Figure 2) in the memory reports, and it did not occur in the controls for theme coverage, *t*(8) = 1.87, *p* > 0.05, Cohen’s *d* = -0.18 or theme reference, *t*(8) = 0.63, *p* > 0.05, Cohen’s *d* = 0.16.

The qualitative analysis of the memory reports and the focus group for the “critical memories” theme informed us about the particular details participants recalled after reading the book *Radical Love*. The reading helped participants deepen their thinking into violent events in the relationship, helped them characterize the relationship as IPV, and increased their awareness of the type of relationship they had experienced.

One of the participants, Eva, shared her memory of a one-night encounter. A man with whom she had intercourse after he pressured her and ignored Eva the day afterward, disseminated a lie on social media about what had happened that night, identifying Eva in those messages. Eva was disappointed about what had occurred, particularly with the lying. In her memory narrative, she reported that after some time, the man pressured her to meet, and she accepted.

In what follows, the first part of the memory report written by Eva before she read *Radical Love* is presented:

We met a night during the summer, in a disco. We were both drunk, and we had almost not talked when we engaged in heavy petting. We were feeling very good and decided to go to the beach to be more relaxed. We did not make it, but we had a lot of sex. At sunrise, we decided to go back to the disco. (…).
After that, I didn’t meet him because I didn’t think that he was my type, and *I did not like that he said things that did not happen*. Though finally, *he pressured me to meet*, and my friends encouraged me to do it, and I accepted. (…)
He was handsome, nice, successful with girls, and funny, but he never made me feel comfortable [P #2]

In this first written narrative, Eva refers to the man’s violent behavior only once, not for what happened that night but that he pressured her to meet after that first encounter. In addition, Eva shares her feelings (disappointment, dislike) about the violent behavior of the man toward her: making a story up and disseminating it among her peers. A selection from Eva’s report explaining the same part of the episode, but now after the reading, follows:

That night, our aim was, basically, to have sex with the greatest number possible of guys, in order for us to feel cool, I guess. After engaging in petting with some guys, this one was the last one I had sex with that night. We were feeling good and went to the beach. *To be honest, he pressured me so much to do so*.
*He kept pressuring me to meet*, and, finally, I decided to get together. *One friend told me he was a jerk and not to meet him*. But all the other ones encouraged me to give him an opportunity (…).
*Right now, he is a person that I disdain so much* [P #2]

Here, Eva includes more critical memories. She reports two violent behaviors of the man toward her. First, and most important, Eva shares that the man pressured her so much to have sex. She adds that she is being honest in saying so. Second, she again explains that the man pressured her to meet after that violent sporadic relationship, but in this second version of the memory report, Eva emphasizes the coercive behavior of the man as she adds “kept” when referring to the man pressing her to meet. Third, and importantly, in the post reading version of Eva’s memory report, while she again mentions her friends when explaining the second time she met with the man, she states in the first place that among her friends, there was one who did not encourage her to get together but said that the man was a “jerk.” Afterward, Eva adds that the other friends thought differently and encouraged her to give him a second chance. Four, after the reading, Eva includes in her report that she feels disdain toward that violent man. All these available details after the reading are substantial differences in comparison with the first version of Eva’s memory report, show more explicitly the influence of the coercive discourse, and have important implications for awareness and prevention.

The events reported by the participants while in the communicative discussion group reflected well the influence of the coercive discourse in the sexual affective relationships reported, and contained details of critical memories, with explicit expression of situations of IPV and indicating awareness of those. In one of the moments of the group discussion, the participants focused on situations in which they did not want to have sex, but the guy coerced them. Idoia shared her story in this regard, in which can be observed: (a) a situation of IPV, (b) details about her emotions in the event, and (c) Idoia’s consciousness about her own behavior and feelings in the relationship while being critical about those:

It is assumed that if that person [the partner] is going to be angry if you are not going to do that [having sex] is because *you should not be with that person*. I think *all of us have made that mistake* [having sex under duress], perhaps because of fear of losing him or because, *in fact, you feel comfortable with that person.* [P #03]

Afterward, Nerea intervened building upon Idoia’s reflection. Nerea interprets Idoia’s story through the lenses of the influence of the coercive discourse which presents violent behaviors and attitudes as attractive. The participants manifested their agency to transform these situations; they shared that once they assume the event as it is, violent, and are critical about it, then “you feel so bad” and “absurd” and come to question their remaining in the relationship. In Nerea’s words:

*You do not want to see what is happening because you love him.* After that, *you are aware and you feel so bad*, and you think, fuck! I am absurd; *I do not know why I have been enduring him*. [P #10]

### Emotions Induced by Memory Recall

The question of whether emotions induced by memory recall could change as a consequence of the reading was explored through an analysis of differences between the pre-test and post-test responses for one question on the MQQ (Alea and Bluck, 2007) that asked the following to participants in both conditions: “Did this memory make you feel… (circle one number for each emotion).” Responses were given on a 5-point Likert scale, ranging from not at all (1) to extremely (5), and the emotions explored were happy, afraid, surprised, and angry. Thus, dependent sample *t*-tests were conducted separately for the intervention and control groups. The results (see Table 2) suggested that the emotions induced by the recall of the memories changed between the pre-test and the post-test in both groups. However, the direction of the change was different between conditions for some of the emotions. Participants in the experimental group presented a significant decrease in happiness when recalling the memory after the intervention, *t*(31) = 1.73, *p* < 0.1, Cohen’s *d* = 0.29, and increases in feelings of concern *t*(31) = 1.31, *p* > 0.1, Cohen’s *d* = -0.51, surprise, *t*(31) = 0.63, *p* > 0.1, Cohen’s *d* = -0.10, and anger, *t*(31) = 0.50, *p* > 0.1, Cohen’s *d* = -0.06. Feelings of happiness when recalling the memories increased significantly in the control group *t*(30) = 0.62, *p* < 0.1, Cohen’s *d* = -0.10; concern did not change, *t*(28) = 0.16; *p* > 0.1, Cohen’s *d* = 0.02; and surprise, *t*(30) = 1.28, *p* > 0.1, Cohen’s *d* = 0.19, and anger, *t*(30) = 1.77, *p* < 0.1, Cohen’s *d* = 0.25, decreased, the latter significantly.

**Table 2:** Pre- and post-test differences in emotions induced by memory recall.

	Experimental group (*n* = 32)				Control group (*n* = 31)			
		
Subscale	Pre-mean	SD	Post-mean	SD	Pre-mean	SD	Post-mean	SD
Happy^a,b^	2.91	1.15	2.56	1.24	2.74	1.59	2.90	1.56
Concerned	2.28	1.17	2.44	1.27	2.10	1.32	2.07	0.92
Surprised	2.78	1.34	2.91	1.09	3.06	1.31	2.81	1.19
Angry^c^	2.69	1.33	2.78	1.24	2.61	1.31	2.29	1.24


*^*a*^Experimental group decreased significantly after the intervention. ^*b*^Control group increased significantly. ^*c*^Control group decreased significantly.*

### Memory Sharing and Talk

The MQQ question about sharing and talking with others regarding particular memories related to the sexual-affective relationship was also incorporated in the analysis. Participants were asked: “When was the last time you talked about or shared this memory with others?” The answer was quantified on a 7-point scale ranging from “yesterday” to “never” and included the option of “Other, please describe.” To examine whether there were differences between the pre-test and the post-test in terms of the last time participants had shared or talked about the memory with others, a dependent sample *t test* was performed in every group condition. In the experimental group, the frequency with which memories were shared after the intervention increased significantly, *t*(31) = 2.41; *p* < 0.05, Cohen’s *d* = 0.29, while in the control group, the frequency of memory sharing was unchanged, *t*(30) = 1.66; *p* > 0.1, Cohen’s *d* = 0.17. Thus, the reading encouraged participants to talk about or share their memories with others more (see Table 3).

**Table 3:** Last occurrence of sharing and talking about memory.

	Experimental group (*n* = 32)		Control group (*n* = 31)	
		
Scale	Pre-test number (%)	Post-test number (%)	Pre-test number (%)	Post-test number (%)
Yesterday	1 (3)	6 (19)	3 (10)	4 (13)
A week ago	12 (37)	12 (37)	8 (26)	13 (42)
A month ago	5 (16)	5 (16)	7 (23)	7 (23)
A year ago	6 (19)	2 (6)	0	0
More than a year ago	2 (6)	2 (6)	2 (6)	2 (6)
Never	0	0	1 (3)	0
Others	5 (16)	4 (13)	9 (29)	5 (16)
No response	1 (3)	1 (3)	1 (3)	0


In particular, changes in memory sharing in the experimental group occurred in two main categories, with a number of participants changing after the reading from sharing their memories of the relationship “a year ago” to “yesterday” - that is, the day before the second measurement. In the pre-test, 3% of participants selected the “yesterday” option, and 19% selected “a year ago.” Later, in the post-test, 19% of participants selected “yesterday,” and 7% selected “a year ago.”

In addition, the results from the focus group informed us about *how* the sharing of memories with others occurred. One way participants shared their personal reflections evoked by the reading was through cell phone apps and other social media, as well as by meeting physically. For example, one of the participants said that while she was reading, she shared the main messages from the text with her friends through online chatting on WhatsApp because she realized that the lessons in the book applied not only to herself but also to her female friends who had had similar experiences of violent sexual-affective relationships. After sharing online, they ended up meeting at the participant’s house:

Participant: And while I was reading, I was chatting on WhatsApp with a friend: “Hey! I’m reading this, and this happens to you!,” and my friend said “Oh, keep reading, I would like to know more! Keep sending audios because I don’t understand this part…”
Researcher: So, did you explain it to your friend?
Participant: Yes, and after that, she came to my house, and I told her: “Ok, this situation is similar to your situation, you know, you can’t continue with this, eh… No!”

### Prospective Thinking and Decision Making

In addition, the results from the qualitative analysis of the second version of the memory reports indicated that the experience of reading *Radical Love* (Gómez, 2015) led some participants to make decisions about introducing changes in the relationship if they were still in it, including freeing the relationship from violence and/or leaving the relationship temporarily: “Now I know that I can’t let him treat me like that” [P #17], “I have decided to stop talking to him for some time” [P #29]. Some participants also manifested engaging in new prospective thinking that consisted of avoiding the relationship with that same man at that time or with similar men in the future: “I know that I would never have a relationship with him or with men like him” [P #06]. What is more, one participant decided to break with the relationship weeks after the intervention.

## Discussion

### Reconstructing Memories About Violent Sexual-Affective Experiences to Prevent Gender Violence Among Youth

The coercive discourse, which presents male violent behavior and attitudes toward women as attractive, has been identified in research as one more cause for violence against women (Valls et al., 2008). Our study examined the effectiveness of reading a scholarly text that questions such discourse to reconstruct young women’s autobiographical memories of violent sexual-affective relationships along preventive lines. The findings demonstrate that reading *Radical Love: A revolution for the 21^st^ Century* (Gómez, 2004, 2015) did support changes in the autobiographical memories in two ways. The first was in their semantic and emotional content: the reading produced greater accessibility to and sharing of critical memories, an increase in negative emotions, and a decrease in positive emotions induced by the recall of the memory. Second, the reading increased the talking about and sharing of the memories recalled, and the reconstructed memories fostered prospective thinking that could support the avoidance of violent sexual-affective relationships in the future and even breaking with the violent relationship if it was present by the time of the study.

These results are consistent with central findings on the malleability of autobiographical memories and its social nature; they are subjective remembering of first-person experiences filled with emotion (Conway and Pleydell-Pearce, 2000; Cohen and Conway, 2008). Thus, one can look at the past and remember it differently depending on particular moods, time periods, new objectives of the self, and important life experiences, among other factors (Conway and Holmes, 2004). Our study provided data showing that reading *Radical Love* favored participants’ awareness about the influence of the coercive discourse in sexual-affective life in general, and also in their own past experiences. Upon such knowledge, the young women exercised their agency and decided to revise their memories of sporadic or stable violent sexual-affective relationships, which allowed them to look at their past critically and freely, beyond the lenses of the coercive discourse. The memory reconstruction resulted from this process. Our findings also shed new light on the power of a particular direction in memory reconstruction (which implies decreasing positive emotions and raising critical memories) to prevent gender violence among young women. Apart from confirming the reconstructive nature of autobiographical memories of violent sexual-affective relationships, our study shed light on how one simple intervention, reading *Radical Love* (Gómez, 2015), can scaffold such reconstruction in specific ways, *via* knowledge and human agency which can drive questioning coercive social influences in the interpretation of one’s past, supporting the prevention of IPV, namely, increasing both critical memories of violent sexual-affective experiences and negative emotions induced by the recall of those memories. Moreover, after the experience some participant women freely decided to abandon or take distance from the relationship if it was present by the time of the study and/or planning for a future with relationships with men with non-violent behavior and attitudes.

In line with the “social turn in memory research” (Hirst and Rajaram, 2014), our findings also reiterate the need to account for the social aspects of memory in order to understand human memory more deeply, showing, in particular, the importance of social context in the construction and reconstruction of autobiographical memories of sexual-affective experiences. Literature in sociology and gender studies has shown that attraction patterns are affected by a coercive discourse (Puigvert and Flecha, 2018) in which male with violent behavior and attitudes are presented as most sexually attractive. This coercive discourse often permeates the media, the peer group, and other socialization agents. Nonetheless, social and educational research has indicated that given their social construction, those attraction patterns can be changed with new interactions and social experiences that can drive learning new attraction patterns where desire is linked to dialog and respect. That learning can protect adolescents from gender violence (Gómez, 2015; Puigvert, 2015/2016). The research in the psychology of memory reported here adds to that literature, showing that the weakening of the association between attraction and violence – imposed by the coercive discourse – can also occur in personal memories of violent sexual-affective relationships, which play a crucial role in the development of the self (Conway and Pleydell-Pearce, 2000) and in planning the personal future (Klein et al., 2010). Our study has shown that by engaging in a concrete social experience, i.e., reading a book that empties men with violent behavior and attitudes of attractiveness, autobiographical memories of violent sexual-affective relationships can be revised semantically and emotionally in ways that weaken the connection between attractiveness and violence. The reading made participants move from more positive to more negative emotions when the memory of the relationship was recalled, as well as reading *Radical Love* (Gómez, 2015) supported the accessibility to details of the experience that demonstrated the violent character of the intimate relationship, as well as it raised reference to oneself in terms of adding details about one’s behavior and/or feelings, and/or those of other close ones in relation to the violent episodes and relationship. This notion of memory plasticity as a result of new social stimuli has been well developed in the literature on memory, including neuroscience and biology perspectives (Santiago Ramón, 1894; Bailey et al., 2015), but its application to the prevention of violence against young women is novel, even more, in its application to education.

Specifically, the reading of *Radical Love* supported the accessibility to critical memories of the violent sexual-affective relationship and aided in their disclosure. After reading, participants shared more details (facts and feelings) in their memory reports that informed about the violent or unhealthy nature of the relationship. This did not occur among the controls. The critical episodic details shared involved references to tension, lies, control, pressure, and despising, along with a certain critical awareness about the violent nature of the behavior and/or attitudes of the man in the relationship toward the woman sharing the memory. Although all participants identified as having experienced a negative sexual-affective relationship, as this was a selection criterion of the study, critical memories –as defined in this study – were loose in the first round of reports. This reveals that reading *Radical Love* increased the accessibility to critical memories and raised awareness, showing the dynamic character of autobiographical memories (Wang, 2016).

The self-discrepancy approach also helps interpreting our results. Applied to intimate relationships, self-discrepancy theory (Higgins, 1987) indicates that real-ideal discrepancy supports relationship dissatisfaction (Casad et al., 2015). Our data showed that through the reading, the women in the intervention group accessed and disclosed more details of the unhealthy relationship they had, this making them more conscious about the *real* type of relationship they had as was evidenced in their own words in the memory narratives after the reading as well as in the focus group. Also, the decrease in happiness in the intervention group when recalling the memory after the reading (which did not happen in the control group) and the decisions and prospective thinking that some participants shared after the reading, such as ending the reported relationship and/or deciding not to have similar relationships in the future, can be explained by real-ideal discrepancy regarding the relationship they had. Further examination of the specific interactions among accessibility to critical memories, increased critical consciousness, real-ideal discrepancy, and impacts on the directive functions of autobiographical memories are a promising area of research in the application of memory studies to the prevention and overcoming of gender violence among youth.

After the reading, females in the intervention group felt less happy, more concerned, and angrier when recalling the relationship. The opposite trend occurred among the controls. This finding is meaningful from the perspective of the interactions between memory and emotion. Prior research has shown that emotions and emotional goals experienced at the time of autobiographical retrieval can influence the information recalled (Holland and Kensinger, 2010). Feeling less happy, more concerned, and more surprised with the new recall could influence the recall of more critical memories. Likewise, it could be plausible that recalling critical episodic details and non-positive emotional contents after the reading might enhance feelings of sadness, surprise, and concern. Building on the results of this study, future research could explore the particular connections among critical memories, the emotional content of memories, and the emotions induced by recall. This inquiry could advance our understanding of the relationship between emotions and autobiographical memories of violent sexual-affective relationships, and it could shed some light upon what element (critical memories, emotional memory contents, or recalled-induced emotions) is best to focus on first in terms of memory-based interventions that aim to prevent gender violence from schools.

Additionally, reading *Radical Love* encouraged participants to share their memory with others. Participants in the focus group explained that they shared the lessons from the book and their revised memories with female friends who they thought and/or knew had similar sexual experiences. This did not occur in the control group. The experimental participants explained that they did so because they thought that it could help other women avoiding the same experiences. This result shows that social experiences, like reading on a topic connected to memories that are most central at a particular life stage: love and attraction (Conway and Holmes, 2004), make the *social* (Alea and Bluck, 2003) and the *directive* (McAdams et al., 2001) functions of autobiographical memories of sexual-affective relationships interact. More specifically, our finding points to a new social function of sharing autobiographical memories of violent sexual-affective relationships after reading *Radical Love*: cultivating friendship through female solidarity. Future research could examine with more detail the extent at which this sharing develops or maintain intimacy, teach and inform, and establish empathy with female friends, as other studies have pointed out such outcomes from autobiographical memory sharing in general (Alea and Bluck, 2007) but not for the case of memories of gender violence in particular. Even how such sharing contributes to meaning making in the young women involved in the intervention seems relevant to be further investigated in the light of other studies on personal meaning fueled by one’s commitment to others’ wellbeing (Tellado, 2017; Garcia et al., 2018). Generally, this unexpected result expanded the social impact of the study (Flecha et al., 2015), opening possibilities of transformative memory reconstruction in other young women beyond the study participants.

### Limitations and Future Research

There are limitations in the research reported. One of them is related to the stability of change. The measures of memory (memory reports and questionnaires) were conducted 10 days after the reading material was given to participants. To examine the persistence of change or the degree of persistence, this measurement should be repeated beyond the following 10 days, such as in 1- or 3-month intervals. The availability of such data could reveal more about the power of *Radical Love* to reconstruct emotional memories of violent relationships in the mid- and long term, as we are seeking more stable changes in cognition and emotion that can support prevention of gender violence throughout development. A second limitation might be, particularly for certain researchers in the emotion field, the self-reported nature of the study. It could be questioned whether there was a discrepancy between what the participants expressed that they felt and what they actually felt during their experience of the IPV and during their recall of the memory itself. It could be argued in this sense that the results are affected by social desirability and, more broadly, by the well-examined power of individual motivations on recall (McDonald and Hirt, 1997). Nonetheless, this second limitation could be contested from the autobiographical memory literature itself, which states that what matters most in human memory is the account of a memory that a person gives to herself and to others, as that is how the memory becomes recoded every time the person shares it (Stone et al., 2010). Nevertheless, the two limitations mentioned are addressed in a new research project (CREA, 2017/2019) in which some of the authors of this article are now engaged; it includes a repeated-measures longitudinal study that observes with behavioral and psychophysiological data the sustained impact of various actions of preventive socialization of gender violence upon the transformative reconstruction both of memories of violent sexual-affective experiences and of implicit emotional reactions associated to the recall of those memories. This new project is conducted in high schools and includes, among others, the intervention reported in this article.

A third limitation in our study is that participants in the reading condition also shared their experiences more with friends. While this is a result of the intervention, this sharing could also be in part responsible for the increase in critical memories. The complex ways in which emotion and memory interact have been pointed out before in our discussion (Holland and Kensinger, 2010). Future analyses could explore the relationship between sharing both the content of the book and one’s memories with others, and changes (if any) in critical memories and emotions induced by the recall of autobiographical memories of violent sexual-affective experiences. This directive for future research in the area of autobiographical memory about gender violence is again strengthened by research framed by the “social turn” in memory studies (Hirst and Rajaram, 2014), which indicates that memories are affected (in its coding and retrieval phases) by social interaction, in which verbal communication and dialog with others about memories (Hedrick et al., 2009) seems to play a central role in memory consolidation and construction. According to this area of research, memory scholars should devote more efforts to explore the scaffolding mechanisms of memory (Nelson and Fivush, 2004). Our study, with its findings on the scaffolding role of reading *Radical Love* upon memory, as well as with its results on memory sharing produced by reading address that demand. Pursuing this line of research with longitudinal studies and similar interventions will advance the understanding of the mediated nature of memory and cognition (Vygotsky, 1980).

### Practical Implications

From the point of view of implications for practice, this research was particularly relevant and innovative. A novel meta-analysis of school-based programs implemented in secondary schools that sought to prevent or reduce incidents of dating violence, concluded the need for incorporating skill-building components (De La Rue et al., 2017). Also, other reviews of intervention strategies in education have indicated the necessity to provide adolescents and youth with the tools to be more critical about violence (Aiello et al., 2018), including gender violence in their everyday experiences and respond to them (Eisenbraun, 2007). Our study provides one evidence-based instrument that teachers, school psychologists, and other educational agents can employ to tackle gender violence from schools and which precisely provides students with cognitive tools to better manage and respond to violence in sexual-affective relationships and to be critical with the coercive discourse that much affects their developmental contexts. Additionally, this intervention, while preventive, takes into account that a relevant number of young women in secondary schools and first college years have already experienced sporadic or stable sexual-affective relationships, and some of those are violent. In terms of innovation, the psycho-educational intervention that we studied is also novel because actions to prevent and respond to gender violence among young women from schools have not usually included the perspective of memory reconstruction.

Educationally, our study adds to the line of research on educational actions that foster preventive socialization of gender violence by evidencing the power of reading scientific literature that empties violence of attractiveness for autobiographical memory reconstruction as a vehicle for preventing and reducing violence against young women. Overall, the findings from our study suggest that educational programs addressed to youth and which seek to prevent and respond to gender violence in stable and sporadic intimate relationships can benefit from incorporating memory-based interventions from a reconstructive and transformative perspective. Schools can employ this educational action as part of a program of gender violence prevention and intervention, with the multiple benefits that addressing this problem has for achievement, sense of school belonging and overall mental health of future generations (De La Rue et al., 2017).

## Author Contributions

SR-P and LU-L conducted the research and investigation process. LP contributed to the conceptualization of the study in relation to the Free_Teen_Desire project. LU-L performed the data collection and SR-P conducted the data analysis, with a particular focus on memory. SR-P, LU-L, LP, and EA contributed to the formal analyses and discussion of the data. SR-P, LU-L, LP, and EA collaborated in writing the manuscript, revised it and approved the submitted version.

## Conflict of Interest Statement

The authors declare that the research was conducted in the absence of any commercial or financial relationships that could be construed as a potential conflict of interest. The reviewer RN and handling editor declared their shared affiliation at the time of review.
